# Antepartum Emergency Department Use and Associations with Maternal and Neonatal Outcomes in a Large Hospital System

**DOI:** 10.1089/whr.2023.0072

**Published:** 2023-12-04

**Authors:** Thwisha Sabloak, Lynn M. Yee, Joe Feinglass

**Affiliations:** ^1^ Program in Public Health, Northwestern University Feinberg School of Medicine, Chicago, Illinois, USA.; ^2^ Division of Maternal-Fetal Medicine, Department of Obstetrics and Gynecology, Northwestern University Feinberg School of Medicine, Chicago, Illinois, USA.; ^3^ Division of General Internal Medicine, Northwestern University Feinberg School of Medicine, Chicago, Illinois, USA.

**Keywords:** antepartum emergency department, antepartum inpatient hospitalization, delivery complications, delivery hospitalization, severe maternal morbidity, prenatal care

## Abstract

**Objectives:**

Prenatal care in the United States has remained unchanged for decades, with pregnant patients often experiencing high rates of hospital emergency department (ED) visits. It is unknown how many of these ED visits are potentially preventable with better access to timely and effective outpatient or home prenatal care. This multihospital health system quality improvement study was undertaken to analyze patient risk factors for acute antepartum hospital use as well as associations with adverse maternal and neonatal birth outcomes.

**Methods:**

The retrospective cohort study analyzed electronic health record and administrative data on ED visits in the 270 days before a delivery admission for alive, singleton births at nine system hospitals over 52 months. We use logistic regression to estimate the likelihood of hospital use by patient demographic and clinical characteristics and present the association of acute antepartum hospital use with maternal and neonatal birth outcomes.

**Results:**

Overall, 17.5% of 68,200 patients had antepartum ED visits, including 248 inpatient admissions, with significant variation between hospitals. As compared to non-Hispanic white patients, Hispanic and especially non-Hispanic Black and Medicaid patients had significantly higher odds of acute antepartum hospital use as did patients with preexisting conditions. Birth outcomes were significantly (*p* < 0.01) worse among individuals with antepartum hospital utilization.

**Conclusion:**

Acute antepartum hospital use was concentrated among lower income, minority patients, and those with chronic conditions with significant variation across system hospitals. There is a need for research into innovations in prenatal care that are best at reaching our most vulnerable patients, reducing preventable hospital utilization, and improving birth outcomes.

## Introduction

Although a majority of pregnant people in the United States receive early prenatal care,^1^ our country's prenatal care model has remained almost unchanged since 1930.^2^ Given high and increasing rates of maternal morbidity and mortality and the continued poor performance of the United States on birth outcomes,^3,4^ many have proposed improvements to the antepartum care model that could lead to better maternal and neonatal outcomes.^5–7^

One signal of problems in the antepartum care model is frequent use of acute hospital care during pregnancy. There have been a few US studies examining the scope and breadth of antepartum visits to the emergency department (ED) or inpatient stays. The population-based studies which have previously been conducted in the United States report markedly divergent estimates of antepartum hospital use, ranging between 20% and 57% of patients with at least one ED visit or nondelivery hospital discharge during their pregnancy.^8^ While a recently published Canadian study from Ontario reported only a much lower composite (9.7%) of antepartum and 42-day postpartum hospital use rate, a recent California study found a 30.4% acute antepartum hospital use rate.^9,10^

The reasons for variation in antepartum hospital use are poorly understood. Although ED visits can be lifesaving for some patients, many ED visits are nonurgent and potentially preventable, had the patient had access to timely in-person or remote care.^11^ High ED visit rates could therefore be a “red flag” for insufficient outpatient care and/or suboptimally controlled chronic or pregnancy-specific conditions. This study was designed as the first step needed to undertake subsequent qualitative (*e.g.*, patient and provider interviews) and quantitative (*e.g.*, ED visit code analysis) studies of specific aspects of actual antepartum ED visits and hospitalizations.

This study profiles acute antepartum hospital use rates at our Midwestern, nine-hospital urban/suburban health system. We aimed to determine the extent of antepartum ED visits, the patient and hospital factors associated with acute antepartum hospital use, and the extent to which there were significant associations with birth outcomes. By describing the full range of perinatal maternal hospital use and associated birth outcomes, our results provide a foundation for future quality improvement efforts designed to reduce what can be stressful, costly, and inefficient care for our pregnant patients.

## Methods

This study used linked electronic health record data for all singleton, live birth deliveries at nine Chicago metropolitan area system hospitals during the 52 months between January 1, 2018, and May 2, 2022. Study hospitals were categorized as one very large urban academic medical center with >12,000 deliveries per year, three large suburban hospitals, and four much smaller exurban community hospitals. Each of the system hospitals have distinct approaches to obstetric triage where pregnant patients may be seen for labor or nonlabor complaints. At the large academic center, most pregnant patients may be seen by specialized obstetric providers emergently in a labor and delivery unit while at other institutions, patients may present directly to the hospital's ED.

The study included all admissions of chronologically first, singleton, live birth deliveries of unique patients, excluding subsequent deliveries among the same patients, in the study period. We excluded deliveries for multiple gestation or if gestational age was below 23 weeks or above 43 weeks. Patient data were derived from their delivery admission and linked to any ED visits as well as antepartum admissions in which a pregnant patient was admitted and discharged without delivery in the 270 days before a delivery admission, whether or not that antepartum admission came through the ED. The study was approved by the Northwestern University Institutional Review Board.

### Patient clinical and sociodemographic characteristics

Delivery admission International Classification of Diseases—Version 10 (ICD-10) diagnosis and procedure codes were utilized to identify vaginal versus cesarean deliveries and previous caesarean delivery.^12^ Maternal age was categorized as ≤19, 20–24, 25–29, 30–34, 35–39, and ≥40 years. Patient race and ethnicity were categorized as non-Hispanic White, non-Hispanic Black, Hispanic/Latinx, Asian, and other/unknown, based on patient self-reported data from delivery admissions. Preferred language was categorized as English versus non-English. Insurance status was categorized as Medicaid or private/other. Patient zip codes were matched to data from the 2020 American Community Survey (ACS) 5-year Zip Code Tabulation Area (ZCTA) file for the percentage of households living at or below the federal poverty level, categorized as <5%, 5%–9.99%, 10%–19.99%, >20% poor households, or being non-Illinois residents.^13^ Year of the delivery admission was coded as 2016–2022.

Following methods used in previously published studies of birth outcomes,^14^ we used data from the delivery admission to characterize the prevalence of any chronic condition from ICD-10 codes for cardiac disease, bleeding disorder, pulmonary hypertension, chronic renal disease, gastrointestinal disease, HIV/AIDS, bariatric surgery, asthma, connective tissue or autoimmune disease, neuromuscular disease, and thyrotoxicosis;^15^ anemia was separately categorized as it had a much higher prevalence. While we include antepartum preeclampsia, it should be noted that when detected in the ED, most patients may not have been discharged, and if admitted into the hospital before delivery, those ED visits which resulted in an inpatient stay delivery admission would not count as antepartum hospital use. Data on patient body mass index (BMI) at birth was categorized as normal weight (≤23.99 kg/m^2^), overweight (25–28.9 kg/m^2^), obese (30–33.9 kg/m^2^), and BMI of 34.0 or greater, or missing). We also coded a history of depression or severe mental illness, as well as substance use. We found that hospital ICD-10 coders had coded *both* gestational and preexisting diabetes and hypertension for a substantial number of delivery admissions; we therefore characterized diabetes and hypertension codes as either uniquely preexisting or gestational, or ICD-10 coded for both.

### Maternal and neonate outcomes

Maternal delivery outcomes included severe maternal morbidity, as defined by Centers for Disease Control ICD-10 codes, vaginal delivery complication (hemorrhagic, infectious, and thrombotic) or cesarean delivery complication (infectious, hemorrhagic, operative complications, and thrombotic).^15^ Long length of delivery admission stay was defined as a length of stay greater than 4 days for vaginal delivery or greater than 6 days for cesarean delivery. We also linked data to postpartum ED visits or inpatient admission in the 90-day postpartum period, which would include most pregnancy-related complications. For neonatal outcomes, low birth weight was defined as <2,500 g, macrosomia as >3,999 g. Neonatal outcomes included preterm birth, defined as gestational age at birth before 37 weeks, and whether the neonate required a neonatal intensive care unit (NICU) admission or had a 5-minute Apgar score <4.

### Statistical analysis

Our primary analysis was factors associated with acute antepartum hospital use, our secondary analysis was the strength of associations between acute antepartum hospital use and maternal and neonatal birth outcomes. The bivariate significance of associations between acute antepartum hospital use and sociodemographic and antepartum clinical and hospital characteristics as well as maternal and neonatal outcomes was determined from chi square tests. Multivariable logistic regression of the likelihood of antepartum hospital use was used to determine the magnitude and statistical significance of associations with patient and hospital characteristics described above. All analyses used Stata, version 16 (StataCorp LLC, College Station, TX, USA).

## Results

**FIG. 1.**
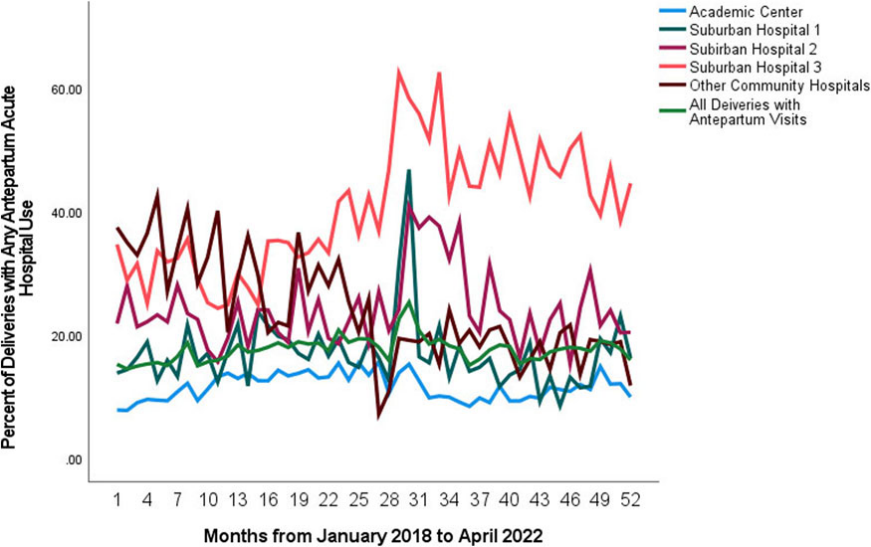
Monthly percentage of deliveries with antepartum acute hospital use by unique patients with Singleton, Live Deliveries at Chicago Nine Metropolitan Chicago Area Hospitals (*N* = 68,200), January 2018 to May 2022.

From January 2018 to May 2, 2022, 68,200 patients had a live, singleton delivery. Overall, 11,717 patients had an ED encounter and 248 had an inpatient admission, for a total of 11,965 patients (17.5%) of sample deliveries with acute antepartum hospital utilization. Figure 1 shows monthly acute antepartum hospital use trends from 2018 to the end of the study period in May 2022, by hospital. Apart from the academic center, which includes over 60% of the study deliveries, there was a sharp increase in ED visits at other system hospitals immediately after the end of the statewide pandemic shut down (month 28–29), during reopening in June 2020 (month 30). Suburban hospital three showed by far the highest rates of acute antepartum hospital use which increased to over 40% of all deliveries after the pandemic shutdown in March 2020.

Table 1 presents both bivariate associations with acute antepartum hospital use and multivariable odds ratios (ORs) for the likelihood of any antepartum hospital use by patient and hospital characteristics. As seen in Table 1, acute antepartum hospital use rose throughout the study period from 15.7% in 2018, peaking at 19.2% in 2020 before declining in 2021–2022. The academic center, which utilizes a dedicated obstetric triage system, had only an 11.6% antepartum hospital use rate, as compared to a 39.0% rate at one of the suburban hospitals. Patients with two or more previous pregnancies had an 8.0% higher incidence and 23% higher odds of an acute antepartum hospital visit as compared to nulliparous patients. Although the proportion of acute antepartum hospital use was lowest among patients in the 30–39 age range, after controlling for the effects of comorbid conditions in regression analysis (highly concentrated among the older patients), both the youngest and the oldest patients had significantly higher adjusted odds of acute antepartum hospital use than the 25–29-year-old reference group. The 5.4% of the sample who spoke a language other than English had significantly lower acute antepartum hospital use.

**Table 1.** Bivariate Associations and Multiple Regression Results for the Likelihood of Any Acute Antepartum Hospital Use by Patient Sociodemographic, Clinical, and Hospital Characteristics

	Percent all births (*N* = 68,200)	*Percent acute antepartum hospital use (*N* = 11,965)*	Odds ratio (95% confidence interval)
All deliveries	100	17.5	
Year of delivery			
2018	24.7	15.7	Reference
2019	24.6	18.3	1.22 (1.14–1.29)
2020	23.1	19.2	1.32 (1.24–1.41)
2021	21.4	17.0	1.10 (1.03–1.17)
(through May 2) 2022	6.2	17.7	1.13 (1.03–1.25)
Hospitals			
Urban Academic Medical Center	60.8	11.6	Reference
Suburban Hospital 1	8.9	16.9	1.50 (1.39–1.62)
Suburban Hospital 2	13.6	39.0	2.32 (2.15–2.52)
Suburban Hospital 3	6.8	23.9	4.77 (4.50–5.05)
Other four community hospitals	9.9	20.8	1.62 (1.50–1.74)
Parity			
Nulliparous	53.3	15.0	Reference
Parity 1	28.4	18.9	1.16 (1.11–1.22)
Parity 2+	18.3	23.0	1.27 (1.19–1.35)
Maternal age			
<20	1.3	22.6	1.20 (1.00–1.43)
20–24	8.1	22.8	1.21 (1.11–1.32)
25–29	19.8	18.7	Reference
30–34	40.6	16.0	1.03 (0.97–1.10)
35–39	24.8	16.8	1.11 (1.03–1.18)
40+	5.4	19.2	1.25 (1.13–1.39)
Race and ethnicity			
Non-Hispanic White	56.0	17.9	Reference
Non-Hispanic Black	8.8	25.1	1.66 (1.53–1.80)
Hispanic	19.1	18.4	1.09 (1.02–1.16)
Asian	7.8	12.3	0.81 (0.74–0.89)
Other/unknown	8.3	10.0	0.67 (0.61–0.74)
Non-English language	5.4	13.3	0.65 (0.59–0.73)
Insurance status			
Medicaid	19.3	23.2	1.30 (1.22–1.39)
Private/other	80.7	16.2	Reference
ZCTA % poverty households			
<5%	47.1	18.2	Reference
5%–9.99%	30.3	17.1	0.95 (0.90–1.00)
10%–19.99%	15.1	17.9	1.03 (0.96–1.11)
20%+	5.1	15.9	0.94 (0.84–1.05)
Not IL Residents	2.4	11.8	0.75 (0.64–0.88)
Maternal BMI (kg/m^2^) at delivery			
Missing	8.1	23.2	1.00 (0.90–1.10)
≤24	9.5	14.1	Reference
25–29	34.4	14.8	1.01 (0.93–1.09)
30–33	23.0	16.9	1.04 (0.95–1.13)
34+	25.0	21.4	1.15 (1.06–1.26)
Prior cesarean delivery	16.3	19.8	1.01 (0.94–1.08)
Preexisting chronic conditions			
Anemia	9.8	23.7	1.32 (1.23–1.41)
Diabetes	1.4	27.2	1.30 (1.11–1.52)
Hypertension	4.8	26.3	1.15 (1.05–1.26)
History of depression or serious mental illness	30.1	25.5	1.71 (1.64–1.79)
Substance use disorder	3.6	41.0	1.94 (1.77–2.12)
Other preexisting chronic conditions^a^	12.3	27.2	1.74 (1.65–1.85)
Pregnancy-related conditions			
Gestational diabetes	5.8	21.8	1.26 (1.15–1.37)
Gestational diabetes for those with preexisting diabetes	1.8	28.9	1.51 (1.31–1.73)
Gestational hypertension only^b^	4.4	17.2	1.06 (0.96–1.18)
Hypertension for those with preexisting Hypertension	1.9	26.1	1.28 (1.11–1.47)
Preeclampsia	0.8	23.6	0.75 (0.60–0.94)

^a^ Preexisting conditions included cardiac disease, bleeding disorder, pulmonary hypertension, chronic renal disease, GI disease, HIV/AIDS, bariatric surgery, asthma, connective tissue or autoimmune disease, neuromuscular disease, thyrotoxicosis, neuromuscular disease.

^b^ All bivariate comparison for acute antepartum hospital use, *p*-value <0.001 except gestational hypertension only.

BMI, body mass index; GI, gastrointestinal; ZCTA, Zip Code Tabulation Area.

As compared to non-Hispanic white patients, Hispanic and especially non-Hispanic Black patients (OR = 1.66, 95% confidence interval 1.53–1.80) had significantly higher likelihood of acute antepartum hospital use while Asian patients and those of other or unknown race and ethnicity had lower odds. Patients with Medicaid were 30% more likely than privately insured patients to have had acute antepartum hospital use. After controlling for all other factors, there were no significant differences by ZCTA poverty level except for lower odds for non-Illinois residents.

Preexisting and chronic health conditions were all associated with higher odds of having acute antepartum hospital use. Patients with a history of depression or serious mental illness (over 30% of the sample), substance use, or a chronic comorbid condition had the highest likelihood of acute antepartum hospital use. Among pregnancy-related conditions, the 4.4% of those coded with gestational hypertension, but not chronic hypertension, did not have significantly different odds of antepartum hospital use and those with preeclampsia had significantly lower use. Patients with mental health, substance use, or serious chronic conditions were between 72% and 93% higher odds of acute antepartum hospital use.

Table 2 presents the associations between acute antepartum hospital use and adverse maternal and neonatal outcomes. Column one shows the incidence of each complication in the study sample, and column two shows the proportion of patients with each complication who had any acute antepartum hospital use, as compared to the overall rate of 17.5%. Patients with maternal complications were significantly (*p* < 0.001) overrepresented among those with acute antepartum hospital use, ranging from 21.2% to 27.0% for delivery complications to a remarkable 46.0% among the 6.7% of patients with 90-day postpartum ED visits or admissions. Higher acute antepartum hospital use was significantly associated with preterm birth and low birth weight, but was not significantly related to macrosomia, NICU admission (*p* = 0.01), or low Apgar scores.

**Table 2.** Association of Adverse Maternal and Neonatal Birth Outcomes with Acute Antepartum Hospital Use

	Sample percent (*N* = 68,200)	Percent of those with complication with acute antepartum hospital use (17.5% in overall sample)
Maternal outcomes		
Severe maternal morbidity^a^	1.4	27.0
Vaginal delivery complication^b^	14.5	21.2
Cesarean delivery complication^c^	18.6	22.3
Long length of stay^d^	3.2	23.2
90-Day postpartum acute hospital use^e^	6.7	46.0
Neonatal outcomes		
Low birth weight	5.6	20.2
Macrosomia^f^	7.9	17.4
Preterm gestational age	7.0	21.9
NICU admission^f^	7.4	18.9
Low Apgar Score^f^	0.5	18.5

^a^ Severe maternal morbidity was defined as hypertensive disorders of pregnancy, infectious disease, nonhypertensive cardiopulmonary disease, or the requirement of a transfusion.

^b^ Vaginal delivery complications were hemorrhagic, infectious, or thrombotic.

^c^ Cesarean delivery complications were infectious, hemorrhagic, thrombotic, or operative complications.

^d^ Length of stay greater than 4 days for vaginal delivery, greater than 6 days for cesarean section deliveries.

^e^ Postpartum ED visits and admissions.

^f^ All associations *p*-value <0.001 except macrosomia, NICU admission (*p* = 0.01) and low Apgar score.

NICU, neonatal intensive care unit.

## Discussion

This study found a 17.5% overall rate of acute antepartum hospital use, which varied significantly across system hospitals. Patients at extremes of age, patients who identified as non-Hispanic Black or Hispanic, and those with Medicaid coverage were at highest risk. The significantly higher likelihood of acute antepartum hospital use by non-Hispanic Black and Hispanic patients reflects long-standing structural racism which has resulted in segregated, high poverty neighborhoods in Chicago, and to some extent, in largely immigrant communities in suburban Cook and DuPage counties. The more frequent use of the ED by pregnant patients from these neighborhoods reflects the lack of prepregnancy health insurance *and* access barriers to ambulatory care (especially at night and on weekends). As expected, acute antepartum hospital use was also higher for those with preexisting or gestational medical conditions, particularly those with mental health and substance use challenges. Maternal delivery outcomes were associated with higher acute antepartum hospital use. In particular, patients who used the ED during pregnancy also had very high 90-day postpartum ED use. Low birth weight and preterm births also had higher antepartum hospital use rates.

The change of antepartum ED visits over the study period seems to have been independent of the spike in visits after the 2020 COVID reopening, which may have been due to the limited availability of in-person prenatal appointments during the April–June 2020 lockdown. While there was generally a return to the previous rates overall after reopening, it was concerning that one suburban hospital continued to have very high rates and there appears to be a moderate but significant overall increase over the study period.

### Previous studies of the incidence of acute antepartum hospital use

Studies based on the chronologically linked hospital data needed to profile episodes of care for pregnant patients are rare. Studies of antepartum hospital use are consistent with our findings of both increased ED visits among lower income and minority patients and worse maternal and neonatal outcomes. However, the literature reflects very widely divergent findings. Several studies of individual hospitals have reported comparatively high rates of ED visits by pregnant patients. Kilfoyle et al. studied pregnant patients delivering over a 2-month period at a high-volume maternity hospital in Rhode Island and found that 84% of pregnant women received care in the ED during their pregnancy, almost a third for nonacute conditions.^16^ Vladutiu et al. studied utilization of emergency care among pregnant Medicaid recipients in North Carolina, finding that 57.5% of pregnant patients sought emergency care before delivering.^17^ Similarly, Ismailova et al. examined ED use during pregnancy among Black women in the Detroit Metro area and found that ∼70.5% of the women had an ED visit at some point during pregnancy.^18^

### Risk factors for acute antepartum hospital use

Although many pregnant patients may have an emergent medical problem that requires prompt ED evaluation, others are likely to choose to use the ED for nonurgent reasons. This is in part driven by the lack of access to outpatient care on weekends or evenings. The Kilfoyle study noted that the two most common reasons for presentation to the ED were concern that there was an emergency (45%) or being referred by a health care provider (36%),^16^ while Vladutiu et al. noted that threatened labor and abdominal pain were the leading indications.^17^ Gilroy et al. conducted a retrospective chart review of patients who received prenatal care in a resident-run clinic in and found the majority of patients had pain (37%) or bleeding (27%) as their presenting complaint.^19^ Monti et al. found that the most common primary diagnoses for antepartum ED visits were threatened abortions (19.6%), urinary tract infections (11.2%), and hemorrhage (9.3%).^9^

It is not surprising that the Ontario Canada study reported the lowest rate of composite postpartum and antepartum ED use in the literature. In the United States, inadequate access to effective prenatal care is the likely driver of avoidable antepartum ED visits. Attending prenatal visits decreases the risk of both adverse obstetrical and birth outcomes (*e.g.*, prematurity, fetal demise, and postnatal mortality).^20^ In contrast, in a study of pregnant patients with a substance-related disorder, antepartum ED use and hospitalizations reflect perceived complications,^21^ including both mental and physical health conditions that the ED is poorly equipped to address. Indeed, Gilroy and Tomczyk^19^ found that visiting the ED did not significantly improve early pregnancy quality of care and Varner et al.^10^ concluded that ED use conferred higher future risk of severe maternal and perinatal morbidity. To our knowledge, there are as yet no evaluation studies of prenatal care interventions designed to impact antepartum hospital use.

### Association of acute antepartum hospital use with birth outcomes

Our study demonstrated worse maternal health outcomes for patients with antepartum ED visits, which is consistent with the higher likelihood of visits by those with chronic or gestational conditions. Monti et al. linked hospital administrative data from 246 California hospitals and found patients with any antepartum hospital use, and particularly those who were admitted for inpatient stays, were significantly more likely than patients without antepartum hospital use to experience a delivery complication, even after controlling for conditions coded during the delivery admission.^9^ Varner et al. examined hospital birth data in Ontario and found vaginal or cesarean delivery complications, or long length of stay after delivery (>4 days for a vaginal delivery and >5 days for a cesarean delivery) was higher among those with a prepregnancy ED visit than those without (22.3 vs. 16.5 per 1,000 births).^10^ They also found a dose–response relationship between antepartum ED use and an increasing risk of adverse maternal outcomes.

### Implications for quality improvement in prenatal care

Decreasing ED use during pregnancy can prevent unnecessary diagnostic and treatment which can cause patients to experience heightened stress and anxiety, and results in excess cost.^20^ Unlike our academic hospital, which has specified obstetric triage providers, many community hospitals utilize the ED for nonlabor complaints, and ED providers in those institutions typically do not have specialized professional training in caring for pregnant people. Obstetric triage promotes faster response to urgent situations, improves maternal and fetal care and bed utilization, avoids unnecessary admissions, reduces the waiting time and patient's stay, increases patient satisfaction and the efficiency of departments while reducing their costs, and reduces maternal mortality.^22^ However, maintaining an obstetric triage unit that is distinct from a labor and delivery unit requires a large and well-staffed obstetrics department, which is why it is generally restricted to large institutions.

### Ideas for redesigning prenatal care

Several new models of prenatal care have been proposed. Peahl et al. suggest (1) designing care delivery around essential services, using in-person care for services that cannot be delivered remotely and offering video visits for other essential services, and (2) creation of flexible services for anticipatory guidance and psychosocial support that allow patients to tailor support to meet their needs through opt-in programs.^23^ Duryea et al. suggest integrating more virtual or audio-only prenatal care visits which can make prenatal care more accessible.^24^ ACOG has even suggested group prenatal care models designed to improve patient education and include opportunities for social support while maintaining the risk screening and physical assessment of individual prenatal care. Bringing patients with similar needs together for health care encounters would increase the time available for the educational component of the encounter, improving efficiency and reducing repetition.^25^ Other models suggest integrating patient navigators, nonhealth professionals such as community health workers, into care teams for higher risk patients.^26^ Navigators can assist patients in attending prenatal care (for instance by assisting with scheduling and transportation), supporting self-management and activation with education about how to handle symptoms and conditions, and often performing social care interventions with connections to community resources. A next follow-up step in this research program is to determine, in consultation with each hospital's clinicians, how best to implement a complexity screening algorithm that can be used to allocate appropriate clinical and social care resources at the first prenatal care visit.

### Study limitations

This study did not attempt to analyze the nature of antepartum ED visits or hospitalizations which inevitably include both pregnancy-related and other diagnoses. Nor did we attempt to determine the preventability of such visits. This retrospective exercise is fraught with uncertainty because presenting complaints (not ICD-10 coded), which would fully justify the urgency of a visit, may not match coded discharge diagnoses which would imply a nonacute visit.^27^ Reasons for ED use conflate lack of 24-hour access to primary office and home care, severity of symptoms, and very specific care processes in use in our large and diverse health system serving a vast urban, suburban, and exurban population across nine-county northern Illinois. This research was undertaken to provide a necessary background for developing locally focused quality improvement projects, including ethnographic research that can illuminate the social and clinical complexities driving hospital use during pregnancy. Another major limitation is that we have no way to ascertain the extent to which patients made visits to other hospitals. Our focus on live singleton births also limits our findings as other patients may have an even higher burden of antepartum hospital use.

## Conclusions

Our study was undertaken to identify the magnitude of acute antepartum hospital use and the patient populations with highest risk. Our next step is to further analyze the reasons for hospital variations in ED use by pregnant patients within our hospital system. We will have to explore the specific reasons patients decide to make ED visits, using mixed methods and deeper patient and provider insight into which visits are potentially preventable. Finally, we need to analyze the extent to which ED use improves or exacerbates adverse maternal or neonatal outcomes.

## Abbreviations Used

BMI: body mass index
ED: emergency department
ICD-10: International Classification of Diseases—Version 10
NICU: neonatal intensive care unit
OR: odds ratio
ZCTA: Zip Code Tabulation Area
